# Practice of Medicine and Pathology

**Published:** 1866-01

**Authors:** 


### PRACTICE OF MEDICINE AND PATHOLOGY.

### *Cholera—Is it Transmissible?*

Every physician must feel the importance of this question, particularly at a time when the authorities of every port along our wide seaboard are called upon to take decided action in the matter of quarantine, and naturally look to our profession for advice. Unfortunately, it is a question concerning which opinions widely differ. The majority of physicians in New England, perhaps, believe that it is not contagious, but mostly, so far as we know, on evidence of a negative character. Dr. Snow, the Superintendent of Health in Providence, in a recent tract for the people, says that it would be a "work of supererogation" to attempt to prove that it is not contagious, and that no person of intelligence can believe otherwise. The evidence he offers is also wholly of a negative character. As may be supposed, he considers quarantine wholly unnecessary, and assumes that the medical faculty of Paris have the same faith. With no evidence of our own to offer, we shall again present to our readers the views of those who have had fresh opportunity of observation, and it will be seen how strong is the belief which prevails among those who are now witnesses of its ravages in its contagious nature. Indeed, not a dissenting voice is raised to such opinions as these which follow in the discussions at the medical societies, which fill the pages of foreign journals.

At the session of the Academie des Sciences, Nov. 6th, M. Velpeau read a paper of some length on the subject of the cure of cholera. He believes the disease is contagious, and states

"that all observations made upon the appearance of cholera in those places where it is not epidemic—for example, in Western Europe—seem to me to prove, almost to a certainty, that cholera is contagious."

The following extract is translated from the *Archives Generales* of November. It is a portion of a memoir presented to the Academie de Medecine by M. Jules Worms, Medecin en chef de l'hopital militaire du Gros-caillou.

"There is a special agent produced upon the banks of the Ganges, and under circumstances but little understood, which is poisonous to many persons.

"This agent manifests itself among persons living or traveling together, but always presents an uninterrupted chain.

"The cholera is a disease transmissible by men.

"This agent manifests its influence upon certain individuals of the human species (probably, also, upon certain kinds of animals,) by effects more or less grave.

"The proportion of persons who are affected by this agent can only be approximately estimated, and is always very small. The human organization may become a fertile soil for the multiplication of this agent, as soon as it shows its poisonous effects.

"The multiplication of this agent takes place particularly in the digestive canal.

"The alvine and gastric discharges of patients affected with cholera contain the effective agent in transmission.

"This efficacy does not coincide with the escape of the discharges. It is subsequent to them by several days.

"It appears to be destroyed after a space of from fifteen days to three weeks.

"The corpses of cholera patients emit the poisonous agent in a much higher degree than the sick.

"Persons affected only with cholerine emit with their dejections an agent capable of producing true cholera about them.

"The greater or less density of the sun's rays to which these dejections are exposed diminishes or favors the propagation of the disease.

"The circumstances which, besides the individual receptivity, the conditions of which are entirely unknown, favor the effectual reception of the toxical agent, are the gastro-intestinal affections, depressing affections of the nervous system, errors of diet, excesses, and all things which diminish the organic energy necessary for the elimination of the poisonous agent.

"Its energy is in proportion to its concentration, and this to the importance of the foci.

"The radius of effectual action of this agent is very limited.

Its diffusion in the atmosphere diminishes and annuls its effects.

"The practical indications which flow from these conclusions are as follows:

"The establishment of certain measures with regard to healthy persons and objects coming from infected places.

"The state of science ought to convince us that healthy persons and objects which have not been used by the sick are little adapted to bear the poisonous agent. This, to be efficacious, must be produced in quantity, (as this only happens among the sick, and is fixed upon objects which have received their dejections.)

"Very rigid measures with regard to sick persons arriving from an infected country, by isolating the sick and by the disinfection or destruction of their dejections, the disinfection of places occupied by them. The measures applied with care in other countries have produced unlooked-for results.

"Well-regulated sanitary inspection. An appeal to all the physicians of the country to announce to the authorities the first cases of the disease, and to apply to the first patients and their dejections the preceding rules.

"The necessity of not leaving in the houses, but of transporting to special places, the dead bodies.

"Isolation of the sick.

"Never to forget that even in a pronounced and extended epidemic the barriers opposed to the radiation of every case of cholera in particular, may prevent numerous misfortunes.

"To remember the facts which have been observed of the recrudescence of epidemics in the spring after being quieted by frost, and to take measures to escape the possible subsequent ravages."

The following extracts are translated from the memoir communicated by M. Grimaud to the Academie des Sciences, entitled "Etudes sur la Cholera faites a Marseille en Septembre et Octobre, 1865:"

"*Origin of the Epidemic.*—Sunday, June 11th, at half-past two, the Stella, Capt. Regnier, entered the port Napoleon. The ship had left Alexandria the first of June with 97 passengers, of whom 67 were Algerian pilgrims. She brought the first news of the existence of cholera at Alexandria. On the evening of the same day, June 11th, the Bizantine arrived with 55 passengers, having left Alexandria, June 3d, and touched at Malta. On the 15th, the Syria, with the English mail and 320 passengers, and on the 16th the Said, with 190 passengers.

"Between the 11th and 16th of June, then, 562 persons arrived at Marseilles from Alexandria, where the epidemic was in the ascendant at the time of their departure. What has become of those 562 persons? They are scattered, but if one could call them by name, the tomb would reply for more than one of them.

"But I have been able to follow, step by step, from the time of their arrival at Fort St. Jean to their departure, the destiny of the 67 pilgrims. The ship left Alexandria the first of June, bearing 67 pilgrims from Mecca. Eight days after its departure, June 9th, there were thrown into the sea two of this number, and two days after this, on the 11th, the 65 remaining disembarked, of whom Ben Kaddour succumbed on landing.

"These pilgrims came from Mecca via Djeddah and Suez. From May 20th to June 22d, nearly 20,000, *all more or less infected*, passed Suez, according to the report of the chief physician at the Ismuth, in order to embark at Alexandria for Europe or elsewhere. Between May 20th and June 22d, several thousand of them encamped at Alexandria, near the canal of the Madmoudieh. * * * * * *

"The population of Suez, Alexandria, Marseilles, etc., were healthy, when some pilgrims from Mecca, embarked at Djeddah, came in contact with them, and the cholera, which was at Djeddah, declared itself among them. It was at Djeddah when the Marseilles pilgrims left there. Some of them died during the passage; we know three of them—the two who succumbed a couple of days before arriving at Marseilles, and the third who died on touching shore. The cholera travelled with them; they bore it.

"The Arabs left Fort Saint Jean to re-embark. A crowd of the curious of that populous quarter mixed itself with the pilgrims, surrounded them and assisted them in loading their baggage outside the Fort. It accompanied them along the bridge overlooking that old part of the city. Here it was that the first cases of cholera showed themselves. They were rare at first, and their character misunderstood or concealed. The physicians said, 'let us say nothing about it; it is not necessary to frighten the people.' But the cholera did not remain confined to the quarter where it first appeared; on the 22d of June a frightful case was reported by Dr. Forcade in rue de Rome. Thus it is that the cholera has for the sixth time been introduced and developed in Marseilles.

"*Facts of Contagion.*—I have said that the disease leaves its principle in baggage. I mention no doubtful facts. Here is one of many others:

"Near Sait-Jean-du-Desert, at Saint-Pierre, not far from Marseilles, in an isolated place, a peasant died of cholera; his wife also died. He had not left the country, 'but,' says Dr. Dussiler, 'his wife was a laundress, and had received a bundle of linen belonging to an individual recently arrived from Egypt. It was the husband who opened the bundle and unfolded all the pieces.'

"Still another: The postal department of Marseilles numbers 120 persons, of whom 75 to 80 are clerks; 22 are employed at the bureau of departure and 9 at the bureau of arrival. There has not been a single death, or even a case of sickness at the former bureau, while of the latter nine employees eight have been sick and one has died. These eight have been taken sick one after the other; this has been proved of the first five. The one who opened the despatches from the East fell sick, was '*cholerise*,' was the expression used. Another was put in his place—the same effect, and so on up to the fifth.

"Now all is explained; there is no longer any mystery in the march of the pestilence. The cholera travels with men and with things. Where man and his effects do not travel, there the malady does not show itself. The 562 travelers of the Stella, Byzantine, Syria and Said, with their correspondence and effects, disembarked at Marseilles from the 11th to the 16th of June, are scattered in Europe, and wherever they have settled they have sown the seed of cholera. This seed has germinated wherever it has found a soil prepared for its reception, that is to say, constitutions predisposed by feebleness derived from previous illness, from intemperance, or from the non-observance of the laws of public and private hygiene."—*Boston Medical and Surgical Journal.*

